# Disturbed sensorimotor and electrophysiological patterns in lead intoxicated rats during development are restored by curcumin I

**DOI:** 10.1371/journal.pone.0172715

**Published:** 2017-03-07

**Authors:** Hind Benammi, Hasna Erazi, Omar El Hiba, Laurent Vinay, Hélène Bras, Jean-Charles Viemari, Halima Gamrani

**Affiliations:** 1 Neuroscience, Pharmacology and Environment Team, faculty of Science Semlalia, Cadi Ayyad University, Marrakech, Morocco; 2 Department of Biology, faculty of Sciences, Chouaib Doukkali University, EL Jadida, Morocco; 3 Institut de Neurosciences de la Timone, Unité Mixte de Recherche 7289, CNRS, Université Aix-Marseille, Marseille, France; Central Michigan University, UNITED STATES

## Abstract

Lead poisoning is one of the most significant health problem of environmental origin. It is known to cause different damages in the central and peripheral nervous system which could be represented by several neurophysiological and behavioral symptoms. In this study we firstly investigated the effect of lead prenatal exposure in rats to (3g/L), from neonatal to young age, on the motor/sensory performances, excitability of the spinal cord and gaits during development. Then we evaluated neuroprotective effects of curcumin I (Cur I) against lead neurotoxicity, by means of grasping and cliff avoidance tests to reveal the impairment of the sensorimotor functions in neonatal rats exposed prenatally to lead. In addition, extracellular recordings of motor output in spinal cord revealed an hyper-excitability of spinal networks in lead treated rats. The frequency of induced fictive locomotion was also increased in treated rats. At the young age, rats exhibited an impaired locomotor gait. All those abnormalities were attenuated by Cur I treatment at a dose of 16g/kg. Based on our finding, Cur I has shown features of a potent chemical compound able to restore the neuronal and the relative locomotor behaviors disturbances induced by lead intoxication. Therefore, this chemical can be recommended as a new therapeutic trial against lead induced neurotoxicity.

## Introduction

Lead (Pb) an ubiquitous environmental pollutant [1], causes lead poisonning that remains an important pediatric health problem in both developed and developing countries [2]. Lead can cross the placental and blood-brain barrier to induce neurotoxicity. Pb generates numerous oxidative, genetic, metabolic, and enzymatic damages [3] and causes well documented hematological and gastrointestinal dysfunctions as well as the production of neurological impairments [4]. It is now well accepted that low doses of Pb promotes cognitive and motor deficits in human and animals [5–8]. Pb exposure also affects a variety of neurotransmitter systems resulting in a wide range of adverse effects, particularly in the developing brain and spinal cord [9, 10]. Several studies indicate that Pb affects the monoaminergic system activity during development of adult's central nervous system (CNS) [10–12] and alters glutamate and GABA-ergic systems [13, 14].

All these adverse effects of Pb have led scientists to research possible natural substances from medicinal plants known for their detoxifying and anti-oxidant activities.

Cur I (diferuloylmethane) is a member of the curcuminoid family [15] and the most important constituent (77%), of the standardised extract of dried rhizome of *Curcuma longa* compared to the other compounds such as Cur II (17%), and Cur III (3%). Cur I is a low molecular weight polyphenol generally regarded as the most active constituent [16] used as a spice and food preservative in India, China, and South East Asia [17]. Cur I is known to possess diverse pharmacological activities like antitumor, anti-inflammatory [18], anti-protein-aggregate activities [19], anticancer, anxiolytic and antioxidant proprieties [20, 21]. Interestingly, antioxidant potential of Cur I is due to its free radical scavenging activity and metal binding property associated with the presence of phenolic and methoxy groups on the phenyl ring and 1,3-diketone in its structure [22]. These properties have fascinated several scientists to investigate neuroprotective potential of Cur in experimental models of toxicity and clinical and neuropsychiatric states [23, 24].

In addition, other studies have demonstrated that Cur I administration restores the behavioral impairments in stressed rats [25], improves locomotors deficit in central and spinal monoaminergic alterations [26, 27] and traumatic brain injured rat [28]. Indeed, accumulating animal model and cell culture data show that dietary Cur I is a strong candidate to use in the prevention or treatment of major disabling age-related neurodegenerative diseases like Alzheimer’s disease, Parkinson’s disease, and myocardial ischemia [29, 30, 31].

In the present investigation, we were focused on the assessment of the possible neurotoxic effects of Pb and the related developmental disorders in neonates and young rats, together with the neuroprotective potential of Cur I, due to its highly abundance in *Curcuma longa* rhizomes, against Pb neurotoxicity, by means of appropriate sensorimotor and locomotor devices coupled to an electrophysiological study.

## Materials and methods

### Ethics statement

The experimental procedures were confirmed to the guidelines of the University of Marrakech and the European (Council Directive 86/6009/EEC) and French regulations (Ministry for Agriculture and Fisheries, Division of Animal Rights). Experimental procedures were approved by the Neurosciences-INT Marseille Ethics Committee registered at the National Commission of animal experimentation (authorized No. 71). Efforts were made to minimize the number of animals used. Adequate measures were taken to minimize pain and animal discomfort.

### Animals and treatments

Wistar female rats weighing 250–300 g were mated and Sperm-positive vaginal smears were taken to indicate the first day of gestation. Females rats were individually housed in a temperature-controlled animal care facility with a 12 h light–dark cycle.

The females rats were divided in three groups: 1) control pregnant rats received only distilled water (dams as well descending pups) with *ad libithum* acces to food. 2): Pb-treated dams and the pups (14 females and 14 males) issued from this group during 82 days during both gestation and lactation stages received Pb chronically drinking water in a dose of 3g (lead acetate)/L (distilled water). 3): Pb + Cur I: dams and pups received Pb in drinking water and Cur I concomitantly orally by gavage in a dose of 16g/kg dissolved in olive oil; regarding its lipophilic nature [32]. For the present investigation, after birth, pups issued from each group were studied at two stages: P1-P2 (postnatal day 1 and 2) and young (2 months) ages.

### Chemicals

Cur I (95% total curcuminoid content), lead (II) acetate trihydrate and all other reagents were obtained from Sigma-Aldrich Co. (St. Louis, MO).

### Evaluation of short term effects of prenatal lead exposure and Cur I treatment

4 male and 4 female pups from each group were studied daily at P1 and P2 for two types of sensorimotor responses appearing in this stage [33]. A score of “1” is given to the pup when it manifests the appropriate response for each following test:

**Cliff-drop aversion**: during the test, each pup is placed on the edge of a cliff with the forelimbs partially on and partially off this surface. The mature response is that the animal quickly turns its head and forelimbs and avoids dropping. This test allows us the evaluate the somatosensory function [33].

**Forepaw grasping**: when the inside of one paw was gently stroked with an object, the paw flexed to grasp the object. Forepaw grasping responses reflect the locomotor abilities of the limbs and the development of fine motricity [33].

### Electrophysiological recordings

Pups (P1-P2) from group 1, 2, 3 were anaesthetized by hypothermia until no reflex could be evoked by pinching the tail. They were then decerebrated at a postcollicular level, eviscerated and pinned down on to a petri dish. A laminectomy was performed, and the spinal cord and roots were removed from sacral segments up to T8 –T10. The preparation was then pinned down, ventral side up, in the recording chamber. All dissection and recording procedures were performed under continuous perfusion with the artificial cerebrospinal fluid (concentration in mM): NaCl 130, KCl 4, CaCl2 3.75; MgSO4 1.3, NaH2PO4 0.58, NaHCO3 25; glucose 10, oxygenated by a mixture of O2 and CO2 (95% -5%), the liquid is maintained at a temperature of 24–26°C and the pH was adjusted to 7.4. The preparation was allowed to equilibrate for about 1 h.

Spontaneous activity and locomotor-like activity were recorded from lumbar ventral roots (left/right L2 or L2 and L5) by means of glass suction electrodes. Fictive locomotion was elicited by an application of NMDA ⁄ 5-hydroxytryptamine creatinine sulfate (5-HT) for 30 min (NMDA, 5–10 lm; 5-HT, 10 lm; DA, 50 lm). Electrophysiological data were acquired through a Digidata 1440A interface using the clampex10 software (Molecular Devices, Sunnyvale, CA, USA) as previously described by [34].

Data analysis consisted of rectifying and integrating (time constant 50 ms) the raw extracellular recordings from ventral roots (Clampfit 10.0 software; Molecular Devices). The phase relationships were evaluated on 60-s recordings by means of cross-correlation analysis (Clampfit 10.0 software; Molecular Devices). The quality of the locomotor-related alternations was assessed by the negative correlation coefficient at zero phase lag (center of the cross-correlogram). The mean correlation coefficient (R) value is the average of all r observed in each experiment for a given experimental condition.

Spontaneous activity was analyzed with clampfit10.0 software (Molecular Devices). Event detection was performed with the ‘Threshold Search’ followed by the ‘Burst Analysis’. The frequency and the average duration of spontaneous bursts were calculated.

### Evaluation of long term effects of prenatal lead exposure and Cur I treatment

#### Cat walk gait analysis

Twelve animals at different ages: P22, P40 and P60 from group 1, 2 (3g/L) and 3, were used for the Cat Walk automated gait analysis system (Noldus Information Technology Inc., Leesburg, VA). The required training protocol has been described elsewhere [35, 36, 37, 38]. Briefly, the CatWalk is a highly sensitive device consisting of a 1.3 m long glass plate illuminated on the side by dim fluorescent lighting. In a dark (unlit) room, animals are placed on the walkway and allowed to traverse from one end to the other. Direct contact between the paw and glass surface results in light reflection in the form of illuminated foot-prints. Foot-print images are video-recorded by a camera positioned under the walkway.

Rats are not pre-trained to cross the walkway. Following acclimation, the animals were removed from the box and completed 3 consecutive runs (averaging approximately 15 s/run.). The CatWalk apparatus and goal box were cleaned between each animal. Runs with walking velocities >30% variation were excluded from analysis. The images from each trial were converted into digital signature and processed using CatWalk XT 9.1 software. The mean scores from 3 consecutive trials (per animal/time points) were analyzed for statistical significance.

Following the identification and labeling of each footprint, a wide range of gait data was generated including: i. the static parameters: relative spatial relationship between different paws (base of support and stride length), ii. Interlimb coordination (regularity index) and iii. Dynamic parameters: (stand, stance, single stance, step cycle and walk speed). The meaning of each parameter is described by Hamers et al. [38].

## Statistical analysis

Data are reported as mean ± SEM and subjected to one-way analysis of variance (ANOVA) and Dunnett’s post hoc test. Values of p lower than 0.05 was considered significant. Statistical analyses were performed using the computer software SPSS 10.0 for windows.

## Results

Short (P1-P2) and long term (P22, P40 and P60) effects of prenatal Pb exposures on the locomotor activity were investigated using electrophysiological recordings, behavioral tests and gait analysis. Treatments by Cur I during the prenatal period also reduced short and long term deficits induced by Pb exposure. The values of all our data are available as supplementary material (S1 File).

### Prenatal lead exposure induced weight loss in pups and Cur I gain it

**Fig 1:**
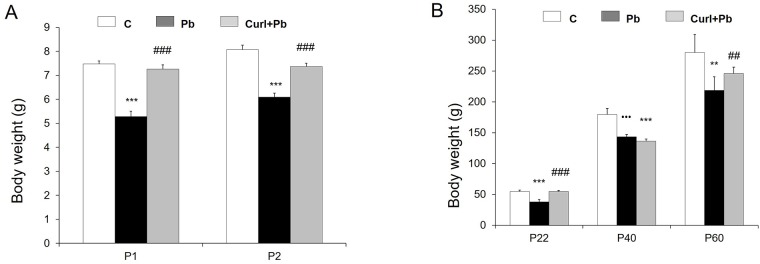
The effect of prenatal lead and Cur I exposure on body weight during development in rat. (A): at (P1, P2) age. (B): (P22, P40, P60) age. * P<0.05 for control vs Pb treated, ^#^ P <0.05 for Cur I +Pb treated vs Pb treated, ^●^P<0.05 for control vs Cur I + Pb treated.

Our data have shown that Pb intoxication (3g/L) reduces significantly (p = 0.004; 0.002; 0.003; 0.004) the pups body weight in comparison to controls, for all ages (P1(5.28±0.22 vs 7.482 ± 0.12), P2 (6.09 ± 0.16 vs 8.07 ± 0.18) (Fig 1A), (P22 (38±3.79 vs 54.57 ± 2.36), P40 (137.4 ± 3.93 vs 170.5 ± 9.63) except for P60 in which the body weight loss is not significant (0.083) (Fig 1B). Treatment with Cur I (16g/Kg) seems to restore the body weight deficiency of the intoxicated group at all developmental stages but more significantly for the young ages compared to Pb group (P1 (7.26 ± 0.17 vs 5.28 ± 0.22), P2 (7.37 ± 0.13 vs 8.07 ± 0.18), P22 (54.8 ± 1.77 vs 38±3.79)) (p = 0.001; 0.004; 0.006).

### Sensorimotor development affected by prenatal lead exposure was restored by Cur I treatment

To reveal the effect of Pb exposure and Cur I treatment during the first four days postnatal (P1-P2) on sensorimotor development, we used appropriate tests such: cliff-drop aversion [33] and grasping [33] tests.

#### Cliff-drop aversion

**Fig 2:**
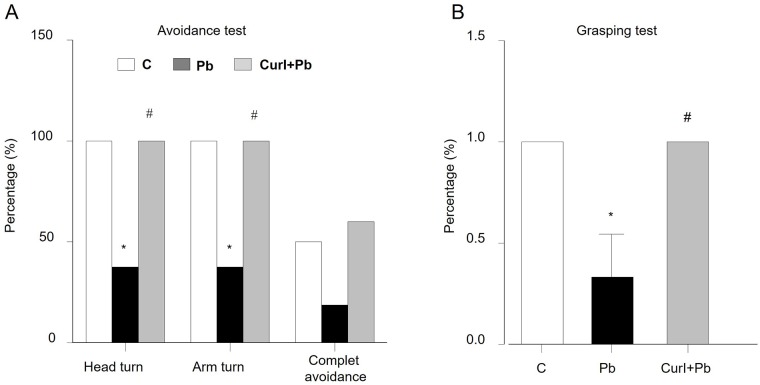
Both prenatal lead at 3g/L and Cur I exposure alters on of labyrinthine function using cliff-drop avoidance task and locomotor abilities of the limbs and the development of fine motility using the grasping task for rat at (P1-P2) age. (A): cliff-drop avoidance task. (B): grasping task *P<0.05 Pb treated vs control.^#^ P<0.05 Cur I + Pb-treated vs Pb-treated.

The test have shown in part, that Pb intoxication reduces significantly the percentage of pups with head (p = 0.01; 0.017) and arms turns (p = 0.01; 0.017) in 37.5% compared to controls and Cur I (Fig 2A).

The percentage of pups that have made a complete drop avoidance in the intoxicated group is lower as compared to control (0.15) and Cur I treated group (0.082) but still not enough significant.

#### Forepaw grasping

The Forepaw grasping responses (dough lightly curved) was significantly (p = 0.051) delayed for Pb-treated rat compared to the controls. While in Cur I+ Pb-treated rats, a significant (p = 0.045) restoration of the Forepaw grasping responses is observed with a return to normal values of control (Fig 2B).

### Lead increased the excitability of spinal networks and effect of Cur I treatment on this abnormality

**Fig 3:**
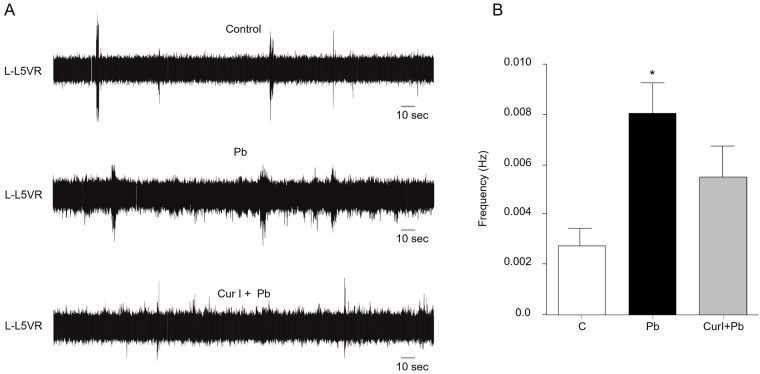
Lead acetate exposure increased spontaneous activity and Cur I restored this impairment for rat at (P1-P2). A: Spontaneous bursts of neuronal activity were recorded from the 5th left (L) lumbar ventral roots after both prenatal exposures to lead acetate at 3 g/L and Cur I for neonates rats. B: Frequency of spontaneous activity. *P<0.05 Pb-treated vs controls.

**Fig 4:**
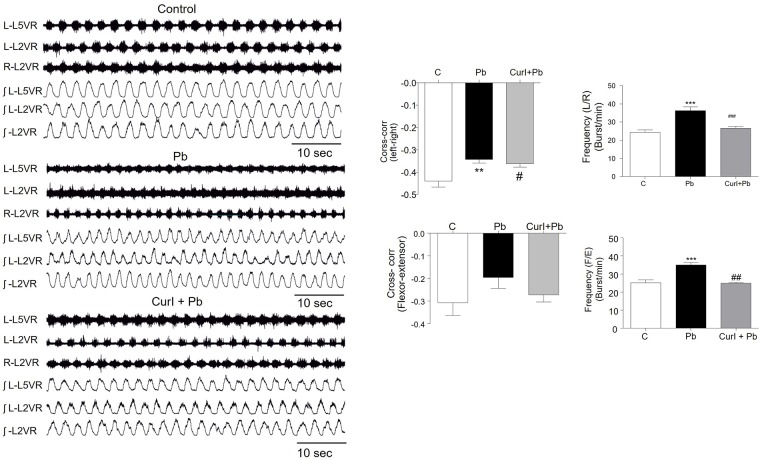
Lead acetate exposure increased fictive activity and Cur I restored this impairment in rat at (P1-P2). (A): electrophysiological recording of fictive activity pattern were recorded from the 2rd and 5^th^ right (R) and from the 2rd and 5th left (L) lumbar ventral roots in control, Pb intoxicated and Cur I +Pb treated pups at P1 and P2. (B): histogram showing the cross-correlation calculated for the opposite ventral root L2 (R). C: the frequency of fictive locomotion recorded from L2 (Flexor) and L5 (Extensor) (F/E) ventral roots. D: histogram showing the cross-correlation calculated for the opposite ventral root L2 (L). E: the frequency of fictive locomotion recorded from L2 (left-right) ventral roots. ***P<0.001;**P<0.01 Pb-treated vs controls; ^###^P<0.001 Cur I + Pb-treated vs Pb-treated; ^#^P<0.05 Cur I + Pb-treated vs controls.

In this study, we evaluated the effect of both Pb and Cur I on the frequency of spontaneous activity recorded on lumbar ventral roots. We have brought evidence that Pb-treatment (3g/L) significantly (p = 0.004; 0.0009) increases the frequency of spontaneous activity recording from L5 ventral root (Fig 3A) compared with controls (Fig 3B), respectively. However, in preparations from animals treated with Cur I, the frequency of bursts of spontaneous activity was not significantly different compared with controls. Application of NMDA and 5-HT on spinal cord preparations induced fictive locomotion characterized by left motion on ipsilateral flexoreral flexor left motion on spinal cord preparations induction (Fig 4A, 0-10min). Locomotor pattern was characterized by negative values of the cross-correlation coefficient that reached the most negative values after 20 min of NMDA/5-HT application. During this period (20–30 min), recordings from L2 and L5 ventral roots showed alternated rhythmic discharges in control and prenatally treated rats (Fig 4A), The frequency of the right and left (L2) (Fig 4C) and flexor-extensor alternations (L2 and L5) (Fig 4E) is significantly higher in Pb-treated group compared to the control (p = 0.0005; p = 0.0002). This alternation was characterized by a negative value of (R) at t = 0 named cross correlation coefficient. The R-value in Pb-treated 3g/L pups was significantly (p = 0.0027) less negative as in controls (Fig 4B and 4D). However, as for spontaneous activity, Cur I restored significantly (p = 0.0001; p = 0.008) the altered pattern of Pb exposed neonatal rats (Fig 4C and 4E).

### Long term effects of lead exposure and Cur I treatment on gait

#### Static parameters: Lead exposure increased the duration of temporal parameters

**Fig 5:**
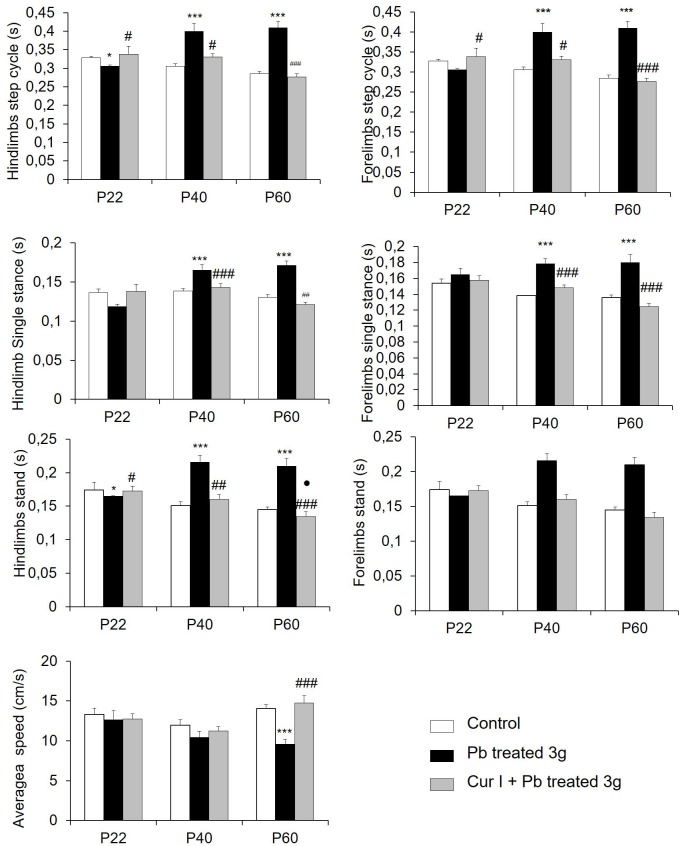
Effect of prenatal both exposure to lead acetate at 3g/L and on general static paw parameters. (A): step cycle (fore and hindlimbs), (B): single stance (fore and hindlimbs), (C): stand (fore and hindlimbs), and (D): average speed. ***P<0.001;**P<0.01 Pb-treated vs controls; ^###^P<0.001 Cur I + Pb-treated vs Pb-treated; ^##^P<0.01 Cur I + Pb-treated vs Pb-treated;*P<0.05 Pb-treated vs controls, ^#^P <0.05 for Cur I + Pb treated vs Pb-treated, ^●^P<0.05 Cur I + Pb treated vs controls.

The difference between the single stance, stand, and step cycle duration is similar in the fore-and hind limbs. Step cycle duration: was increased across in both the fore and hind limbs of the Pb-treated rats at (P40, P60), compared with the control and Cur I+ Pb treated rats at the same ages (Fig 5A). This duration at P22 in Pb-treated rats was shorter than in control and Cur I+ Pb-treated rats (Fig 5A). A similar trend of increased single stance and stand duration was detected in Pb-treated rats compared to the control and Cur I+ Pb group (Fig 5B and 5C). Cur I + Pb-treated rats did not differ significantly from control on temporal parameters. The fore and hindlimbs were identical in all groups and ages.

Pb-treated rats showed significant reductions in walking velocities (average speed) at the three ages studied (P22, P40, P60) compared to the control and Cur I + Pb treated rats (Fig 5D).

#### Dynamic parameters: Lead exposure reduced the dynamic parameters

**Fig 6:**
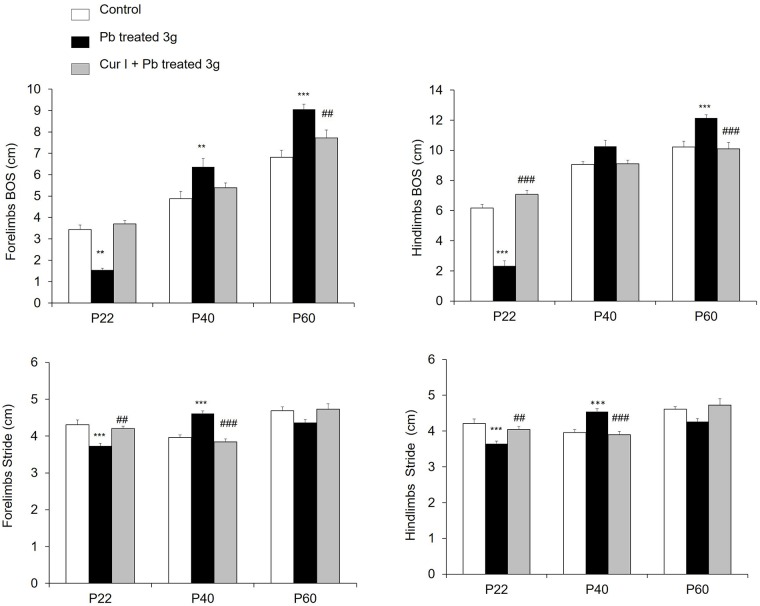
Effect of prenatal both exposure to lead acetate at 3g/L and Cur I on general dynamic paw parameters. (A): Base of support (fore and hindlimbs), (B): stride length (fore and hindlimbs). ***P<0.001;**P<0.01 Pb-treated vs controls; ^###^P<0.001 Cur I + Pb-treated vs Pb-treated; ^##^P<0.01 Cur I + Pb-treated vs Pb-treated.

Base of support (BOS) and stride length were analyzed for both fore-and hindlimbs. In Pb-treated rats the distance between the bilateral paws was significantly (p = 0.009; p = 0.0002) shorter in P22 but higher in P40 and P60 than in control and Cur I + Pb treated groups (Fig 6A). In one side, Pb treated rats had significantly shorter strides in P22 and P60; in other side, they had larger strides in P40 than in control and Cur I + Pb treated rats (Fig 6B). There was no significant difference in BOS and stride length parameters between control and Cur I+ Pb treated rats. The fore and hindlimbs were identical in all groups and ages.

#### Coordination parameters

Regularity index indicates the degree which the animal uses normal step sequence pattern (NSSP). We found, in the present study, no effect of Pb exposure on the (RI) between the three groups (control, Pb-treated and Cur I + Pb treated) at different ages (data not shown).

## Discussion

Pb is one of the most used metals in industrial manufacturers of a wild range of products. Exposure to Pb continues to be a wide spread health problem [39]. The general population may get exposed to Pb due to food and water contaminations, air pollution caused by industrial emission and gasoline containing Pb compound [40].

Several neurobehavioral studies have shown that exposure to Pb have some profound outcomes such as a decreased exploratory behavior in the long run, hyperactivity and altered motor activity [41, 42]. In addition, substantial evidences indicate that brain areas that are involved in motor activity, such as cerebral cortex and basal ganglia, are privileged targets of this metal [41, 43]. Given the vulnerable effect of Pb on locomotor activity shown by different studies already cited, in the present investigation we were focused on the assessment of the possible impairments of the spinal cord network development and sensorimotor behavior occurring in rat developmental as a response of short term (P0-P2) and long term (P 22, P40, P60) Pb exposures and the possible neuroprotective potential of Cur I.

### Short and long effect of Pb on body weight, sensoriomotor and motor coordination

Our data show that administration of Pb via drinking water induces primarily a loss of body weight starting from P1 postnatal age and continues till the young stage. Such effect of Pb has already been described by some authors following chronic Pb intoxication. Canfield and Rezende [44, 45] reports, indeed, a decrease in body weight gain in Pb exposed dams which was attributed to a decrease in food and water intake or the effect of Pb on the synthesis of active metabolites and to intra-uterine alterations [44, 45]. Others sustain a possible interference with glucose metabolism, thus, Pb caused a significant increase of plasmatic glucose levels, which may be induced by circulating adrenaline released from the adrenal medulla and thus may reflect sympathoadrenal medullary activity [46]. Beyond these biochemical abnormalities, Pb is able to affect the neurobehavioral patterns of neonates rats during development. Indeed, the grasping and cliff drop aversion tasks have revealed in the Pb intoxicated pups (P1, P2), respectively a motor alteration of the forelimbs and a delay in sensorial properties. It is well known that grasping task involves a spinal reflex; in addition; fall avoidance enables to evaluate the maturity of sensorimotor functions [47]. This transient motor deficit could reflect a delay in rostrocaudal maturation of the spinal cord. Several studies support our finding. In fact, rats exposed during 3 months to low concentrations (50 ppm) of Pb acetate, resulted in impaired sensory and motor functions using the Post-rotary nystagmus (PRN) [4]. Whereas, previous data have brought evidence of a significant delay of the righting reflex and slant board behavior following Pb acetate (1.0 mM) in drinking water exposure during prenatal and postnatal as well as a latency to fall in the grip strength test [48]. Earlier observations also showed that Pb-treated animals exhibited deficits in righting reflex and grip strength in rats, suggesting a delay in attaining these skills probably because of damage or poor development of the motor system and brain [49].

In order to delineate the neurophysiological basis of the neurobehavioral abnormalities, we performed an electrophysiological study which showed an increased frequency of spontaneous activity recorded from the 5^th^ (L5) lumbar ventral roots indicating a hyper-exitability induced by Pb. In fact, previous data support this observation. Indeed, a study realized by Weinreich [50] showed that Pb exposition to different doses (0, 0.5, 1, and 1.5%), increased the spontaneous activity at the postnatal stage [50]. The spontaneous activity recorded from nerves lumbar engines (ventral roots: the motor outputs) are involved in the development of spinal locomotor circuits [51]. This activity generated prenatally contributes to the neuronal, morphological and synaptic maturations together with the establishment of the electrical properties [52, 53]. On the other hand, our data showed an elevated frequency of left/right rhythmic activity in Pb-treated group indicating a increased fictive activity in those animals. Thus, the cross-correlation coefficient was less negative in Pb-treated group. The same result was obtained for the L3/L5 relationship, reflecting an impaired interlimb coordination. Changes in the frequency of the spontaneous and fictive activities may reflect an imbalance between excitation and inhibition regulated by neurotransmitters such as glutamate, GABA and/or glycine [54, 55, 56]. It’s well known that glutamate is the major excitatory neurotransmitter in the central nervous system [57]. In contrast, glycine (Gly) and GABA are the major inhibitory neurotransmitters [56]. Some studies have demonstrated that glycine and GABA both generated the earliest spontaneous motor activity of fetus and functioned transiently as excitatory transmitters in the embryonic spinal cord at E14.5 [58]. This excitatory hyperpolarization continued around postnatal day 7 (P7) [58]. Several studies indicate that Pb inhibits the potential dependent release of both GABA and glutamate from hippocampal neurons [59]. Moreover, *in vitro* studies have shown that Pb is a potent non-competitive antagonist of NMDA receptors at P21 [60]. Other studies have shown that in the presence of both Pb and Gly, currents activated by NMDA are potentiated; an effect that likely due to the ability of Pb to increase the affinity of the NMDA receptor for Gly [61]. Also application of NMDA agonist blocks spontaneous bursts at E14.5 [58]. All the above findings demonstrate an obvious alteration in spontaneous activity occurring in rats exposed prenatally to Pb. This intoxication affects also the frequency of fictive locomotion known to be generated by specialized neuronal networks called central pattern generators [52]. This activity can be generated pharmacologically *ex vivo* by application of NMDA and stabilized by adding serotonin 5-HT to the bath on spinal cord preparations isolated from neonates induces left/right and flexor/extensor alternations [62]. Previous studies have demonstrated that perikarya of 5-HT neurons in the brain stem project to many regions of the CNS and spinal levels [63]. Thus, an increase of 5-HT innervations from dorsal raphe nucleus (DRN) leads to increased locomotor activity [64]. Such observation was demonstrated previously in adult rat exposed to Pb chronically, showing an increment of 5-HT innervations in the same nucleus [65].

Sensorimotor abnormalities occurring in young pups following Pb administration (our data), were accompanied by a late profound motor coordination abnormalities, even observed in the cat walk test for both dynamic and static parameters. In fact, it has already been reported, in large cohort of animals, that the parameters of stride length, stand, step cycle and regular step patterns are interdependent from locomotor speed [66]. Also further studies demonstrate, in intact animals and humans, that increased locomotor speed is usually associated with a decreased step cycle duration [67], associated logically with a shorter stand duration and single stance. As reported in our data, previous works have brought evidence of a locomotor impairments following Pb exposure. Indeed, in adult rat, chronic [12] and acute [68] exposition to Pb, reduced considerably the walking speed which was associated with a reduction in stance duration [37]. A large base-of-support that can compensate for an instable gait [38] and a decreased stride length finding was observed following contusion and dorsal transection injuries especially in hindlimbs [35,37]. This deficit in gait seen in Pb-treated pups may result from possible monomaminergic impairments. Previous studies in rat demonstrated, effectively that chronic Pb exposure for 1% reduces levels of catecholamines in cerebral cortex, cerebellum and hippocampus, structures involved in the control of several cognitive and locomotor behaviors [10]. Furthermore, chronic exposure to 5g/L of Pb for 3 months reduced the levels of tyrosine hydroxylase (a key enzyme of dopamine synthesis) expression within substantia nigra pars compacta (SNc) and the frontal cortex [65]. This may be indicative of a reduced dopamine levels [10] and/or dopamine turnover [69]. Other studies have reported an increased [9] or unchanged [69] levels of dopamine after Pb exposure, a discrepancy that is probably due to the studied region and/or to different experimental protocols used. It is well established that the dopaminergic system between SNc and the motor cortex is involved in the control of motor activity [70], so the effect of Pb on monoamines may impact on the locomotor behavior. In addition to dopamine, Pb is known to affect the serotonergic system emanating from raphe nucleus and that has an important role in locomotor activity modulation [71]. Substantial evidences reported that exposure to Pb increased the serotoninergic expression in different structures such as frontal cortex, hippocampus, hypothalamus, striatum, SNc, amygdala and cerebellum [9, 72–75]. In addition, increased serotonin levels, might improved locomotion and the interlimb coordination [76]. In our case, data of static parameters (support base of stride lenght) suggested that the increase in serotonin level due to Pb negatively affected locomotor function of the animal, such discrepancy, might result from type of receptors target [41,77,12].

### Cur I restored the neurobehavioral impairments induced by lead intoxication

Beside the neurobehavioral disturbances described in our Pb intoxicated pups, the present study have brought a new evidence of a powerful neuroprotector property of Cur I against Pb neurotoxicity. In fact, Cur I seem to restore the body weight deficit occurring in Pb intoxicated pups progressively from P1 to young stages. Previous data seemed to corroborate such find in rat and mice, in which, treatment with Cur I (100 mg/kg) reversed the loss of body weight induced by 2,3,7,8-Tetrachlorodibenzo-p-dioxin (TCDD) [78, 79, 80]. Such property of Cur I could be due to its possible action on the digestive system including hepatopancreas and intestines. A recent study have shown in *Carassius auratus* (crucian carp) fed with 5g/kg of Cur I an increased body weight, percent weight gain, which was attributed to increased hepatopancreas weight, hepatopancreas protein content, intestinal weight, intestine protein content together with improved hepatopancreatic and intestinal enzymatic activities such as trypsin, lipase, amylase, alkaline phosphatase, gamma-glutamyl transpeptidase and creatine kinase [81]. In human as well, a randomized controlled trial has shown in patients with metabolic syndrome, that 8 week supplementation with Cur I significantly increased serum adiponectin levels and a reduction in serum leptin concentrations [82].

In addition to the body weight, Cur I has shown an ameliorative on the sensory motor parameters especially grasping and cliff avoidance tests altered by Pb in neonates rat. Rare are investigations who addressed such point. Khan et al [83], have reported in Parkinsonian rats induced by 6-OHDA, that Cur I reverses the increased mean time taken to cross a 105 cm beam indicating that the rats following treatment with Cur I have more ability to coordinate their movement on a narrow beam [83]. This effect could imply a possible adjustment of the neuronal network functioning within the spinal cord of pups rats by acting as a Pb chelator, with the possible formation of a complex between the metal and this ligand [84] reducing therefore its bioavailability [85]. Cur I may also act as a neuromodulator of the neuronal firing [86], including the central monoaminergic system by facilitating monoamines release in the brain of mice or rats [87, 88]. Other reports demonstrated that Cur I increased the spinal monoamine (serotonin, noradrenalin) and metabolite (3-methoxy-4-hydroxyphenylglycol (MHPG)) [27]. The enhanced monoamine levels may explain the performance of dynamic parameters shown in the Cur I treated rats. Support of this view, is provided by the fact that Cur I counteracted the locomotor deficit and normalized levels of all plasticity markers in the spinal cord seen in traumatic brain injured animals [28]. The adjustment of the oxidative state could also be suggested as a further mechanism of neuroprotection. Thus, some data showed that Cur I readjusts the oxidative state by acting as a scavenger of reactive oxygen species (ROS) and reactive nitrogen species, therefore, it helps in overcoming enhanced oxidative stress. It also decreases the level of lipid peroxides and augments the activity of antioxidant enzymes such as superoxide dismutase (SOD), catalase (CAT), glutathione peroxidase (GPx), glutathione reductase (GR) and glutathione (GSH) [89]. Those elements are known to be affected by Pb which facilitates the onset of oxidative stress on account of the generation of (ROS) such as hydroperoxides (HO2•), hydroxyl radical (HO•), superoxide radical (O2•), and hydrogen peroxide (H2O2) with a parallel depletion of antioxidant enzymes including GSH, SOD, CAT, GPx, and GR [90, 91].

## Conclusion

Through the present investigation, we have brought evidence of a powerful neurotoxic effect of Pb which impairs different neurobehavioral patterns in neonates rat. Pb neurotoxicity seems to involve an alteration of the locomotors neuronal networks during development resulting in a profound motor deficiency outcome. Treatment with Cur I fascinatingly restores the above abnormalities and may be considered as one of the potent neuroprotective chemicals preventing central nervous system dysfunction during earlier stages of development.

## Supporting information

S1 File The values of the data for the main figures. Table A: The effect of prenatal lead and Cur I exposure on body weight during development in rat (Fig 1). Table B: Both prenatal lead at 3g/L and Cur I exposure alters on of labyrinthine function using cliff-drop avoidance task and locomotor abilities of the limbs and the development of fine motility using the grasping task for rat at (P1-P2) age (Fig 2). Table C: Lead acetate exposure increased spontaneous activity and Cur I restored this impairment for rat at (P1-P2) (Fig 3). Table D: anguished: Lead acetate exposure increased fictive activity and Cur I restored this impairment in rat at (P1-P2) (Fig 4). Table E: Effect of prenatal both exposure to lead acetate at 3g/L and on general static paw parameters (Fig 5). Table F: Effect of prenatal both exposure to lead acetate at 3g/L and Cur I on general dynamic paw parameters (Fig 6).
(DOC)
