# Supplementary material for: Disturbed sensorimotor and electrophysiological patterns in lead intoxicated rats during development are restored by curcumin I
Source: PLoS One. 2017 Mar 7;12(3):e0172715. doi: 10.1371/journal.pone.0172715 (PMC5340392; doi:10.1371/journal.pone.0172715)
Supplement: S1 File — Table A: The effect of prenatal lead and Cur I exposure on body weight during development in rat (Fig 1). Table B: Both prenatal lead at 3g/L and Cur I exposure alters on of labyrinthine function using cliff-drop avoidance task and locomotor abilities of the limbs and the development of fine motility using the grasping task for rat at (P1-P2) age (Fig 2). Table C: Lead acetate exposure increased spontaneous activity and Cur I restored this impairment for rat at (P1-P2) (Fig 3). Table D: anguished: Lead acetate exposure increased fictive activity and Cur I restored this impairment in rat at (P1-P2) (Fig 4). Table E: Effect of prenatal both exposure to lead acetate at 3g/L and on general static paw parameters (Fig 5). Table F: Effect of prenatal both exposure to lead acetate at 3g/L and Cur I on general dynamic paw parameters (Fig 6). (DOC) [file pone.0172715.s001.doc]

**Supplementary data**

**Table A: The effect of prenatal lead and Cur I exposure on body weight during development in rat.**

|  | | | |
| --- | --- | --- | --- |
| **Age** | **Groups** | **Average** | **Standard deviation** |
| **P1** | **C** | 7,48 | 0,12 |
|  | **Pb** | 5,28 | 0,22 |
|  | **Pb+ CurI** | 7,26 | 0,18 |
| **P2** | **C** | 7,37 | 0,14 |
|  | **Pb** | 6,09 | 0,16 |
|  | **Pb+ CurI** | 8,07 | 0,18 |
| **P22** | **C** | 54,57 | 2,37 |
|  | **Pb** | 38 | 3,80 |
|  | **Pb+ CurI** | 54,8 | 1,77 |
| **P40** | **C** | 179,57 | 9,63 |
|  | **Pb** | 143,5 | 3,93 |
|  | **Pb+ CurI** | 136,33 | 3,61 |
| **P60** | **C** | 280 | 29,24 |
|  | **Pb** | 218,66 | 21,90 |
|  | **Pb+ CurI** | 246 | 10,14 |

**Table B: The both prenatal Lead at 3g/L and Cur I exposure alters on of labyrinthine function using cliff-drop avoidance task and locomotor abilities of the limbs and the development of fine motility using the grasping task for rat at (P1-P2) age**

1. **Cliff-drop avoidance task.**

|  |  | | |
| --- | --- | --- | --- |
|  | **Groups** | **Average** | **Standard deviations** |
| **Head turn** | **C** | 100 | 0 |
|  | **Pb** | 37.5 | 0.5 |
|  | **Pb+ CurI** | 100 | 0 |
| **Arm turn** | **C** | 100 | 0 |
|  | **Pb** | 37.5 | 0.5 |
|  | **Pb+ CurI** | 100 | 0 |
| | **Complet avoidance** | | --- | | **C** | 50 | 0.55 |
|  | **Pb** | 18.75 | 0.40 |
|  | **Pb+ CurI** | 60 | 0.55 |

1. **G**rasping task

|  |  | | |
| --- | --- | --- | --- |
| **Groups** | | **Average** | **Standard deviations** |
| **C** | | 1 | 0 |
| **Pb** | | 0,33 | 0,44 |
| **Pb+ CurI** | | 1 | 0 |

**Table C: Lead acetate exposure increased spontaneous activity and Cur I restored this impairment for rat at (P1-P2).**

**(A)Frequency of spontaneous activity**

| **Groups** | **Average** | **Standard deviations** |
| --- | --- | --- |
| **C** | 0,0051 | 0,001 |
| **Pb** | 0.008 | 0.004 |
| **Pb+ CurI** | 0.005 | 0.003 |

**Table D: Lead acetate exposure increased fictive activity and Cur I restored this impairment in rat at (P1-P2).**

**(B) Histogram showing the cross-correlation calculated for the opposite ventral root L2 (R).**

| **Groups** | **Average** | **Standard deviations** |
| --- | --- | --- |
| **C** | -0,31 | 0,38 |
| **Pb** | -0,20 | 0,21 |
| **Pb+ CurI** | -0,27 | 0,22 |

**(C) The frequency of fictive locomotion recorded from L2 and L5 (flexor-extensor) ventral roots.**

| **Groups** | **Average** | **Standard deviations** |
| --- | --- | --- |
| **C** | 24,41 | 3,88 |
| **Pb** | 36,24 | 6,25 |
| **Pb+ CurI** | 30,5 | 24,8 |

**(D) Histogram showing the cross-correlation calculated for the opposite ventral root L2 (L).**

| **Groups** | **Average** | **Standard deviations** |
| --- | --- | --- |
| **C** | -0,44 | 0,10 |
| **Pb** | -0,34 | 0,07 |
| **Pb+ CurI** | -0,36 | 0,04 |

**(E) The frequency of fictive locomotion recorded from L2 (left-right) ventral roots.**

| **Groups** | **Average** | **Standard deviations** |
| --- | --- | --- |
| **C** | 0,005 | 4,37 |
| **Pb** | 0.008 | 3,65 |
| **Pb+ CurI** | 0.005 | 1,91 |

**Table E: Effect of prenatal both exposure to Lead acetate at 3g/L and on general static paw parameters**

**(A) Step cycle (fore and hindlimbs)**

| **Hindlimbs** | | | |
| --- | --- | --- | --- |
| **Age** | **Groups** | **Average** | **Standard deviations** |
| **P22** | **C** | 0,34 | 0,02 |
|  | **Pb** | 0,29 | 0,0004 |
|  | **Pb+ CurI** | 0,31 | 0,01 |
| **P40** | **C** | 0,30 | 0,0008 |
|  | **Pb** | 0,39 | 0,004 |
|  | **Pb+ CurI** | 0,33 | 0,0001 |
| **P60** | **C** | 0,28 | 0,002 |
|  | **Pb** | 0,40 | 0,001 |
|  | **Pb+ CurI** | 0,27 | 0,01 |

| **Forelimbs** | | | |
| --- | --- | --- | --- |
| **Age** | **Groups** | **Average** | **Standard deviations** |
| **P22** | **C** | 0,32 | 0,004 |
|  | **Pb** | 0,30 | 0,003 |
|  | **Pb+ CurI** | 0,33 | 0,03 |
| **P40** | **C** | 0,30 | 0,0005 |
|  | **Pb** | 0,40 | 0,005 |
|  | **Pb+ CurI** | 0,33 | 0,002 |
| **P60** | **C** | 0,28 | 0,007 |
|  | **Pb** | 0,40 | 0,001 |
|  | **Pb+ CurI** | 0,27 | 0,01 |

1. **Single stance (fore and hindlimbs)**

| **Hindlimbs** | | | |
| --- | --- | --- | --- |
| **Age** | **Groups** | **Average** | **Standard deviations** |
| **P22** | **C** | 0,13 | 0.07 |
|  | **Pb** | 0,11 | 0.04 |
|  | **Pb+ CurI** | 0,13 | 0.01 |
| **P40** | **C** | 0,13 | 0,03 |
|  | **Pb** | 0,16 | 0,03 |
|  | **Pb+ CurI** | 0,14 | 0,05 |
| **P60** | **C** | 0,13 | 0,07 |
|  | **Pb** | 0,17 | 0,02 |
|  | **Pb+ CurI** | 0,12 | 0,02 |

| **Forelimbs** | | | |
| --- | --- | --- | --- |
| **Age** | **Groups** | **Average** | **Standard deviations** |
| **P22** | **C** | 0,15 | 0,08 |
|  | **Pb** | 0,16 | 0,01 |
|  | **Pb+ CurI** | 0,15 | 0,07 |
| **P40** | **C** | 0,13 | 0,07 |
|  | **Pb** | 0,17 | 0,09 |
|  | **Pb+ CurI** | 0,14 | 0,04 |
| **P60** | **C** | 0,13 | 0,02 |
|  | **Pb** | 0,18 | 0,01 |
|  | **Pb+ CurI** | 0,12 | 0,02 |

**C) Stand (fore and hindlimbs).**

| **Hindlimbs** | | | |
| --- | --- | --- | --- |
| **Age** | **Groups** | **Average** | **Standard deviations** |
| **P22** | **C** | 0,17 | 0,01 |
|  | **Pb** | 0,16 | 0,07 |
|  | **Pb+ CurI** | 0,17 | 0,01 |
| **P40** | **C** | 0,16 | 0,01 |
|  | **Pb** | 0,22 | 0,03 |
|  | **Pb+ CurI** | 0,16 | 0,06 |
| **P60** | **C** | 0,14 | 0,01 |
|  | **Pb** | 0,22 | 0,05 |
|  | **Pb+ CurI** | 0,13 | 0,05 |

| **Forelimbs** | | | |
| --- | --- | --- | --- |
| **Age** | **Groups** | **Average** | **Standard deviations** |
| **P22** | **C** | 0,18 | 0,03 |
|  | **Pb** | 0,22 | 0,02 |
|  | **Pb+ CurI** | 0,20 | 0,08 |
| **P40** | **C** | 0,15 | 0,01 |
|  | **Pb** | 0,21 | 0,03 |
|  | **Pb+ CurI** | 0,16 | 0,05 |
| **P60** | **C** | 0,14 | 0,02 |
|  | **Pb** | 0,20 | 0,06 |
|  | **Pb+ CurI** | 0,13 | 0,04 |

**D) Average speed**

| **Age** | **Groups** | **Average** | **Standard deviations** |
| --- | --- | --- | --- |
| **P22** | **C** | 13,32 | 3,90 |
|  | **Pb** | 12,68 | 1,99 |
|  | **Pb+ CurI** | 12,75 | 2,21 |
| **P40** | **C** | 11,98 | 2,48 |
|  | **Pb** | 10,44 | 2,21 |
|  | **Pb+ CurI** | 11,22 | 2,30 |
| **P60** | **C** | 14,07 | 1,80 |
|  | **Pb** | 9,59 | 2,37 |
|  | **Pb+ CurI** | 14,76 | 3,76 |

**Table F: Effect of prenatal both exposure to Lead acetate at 3g/L and Cur I on general dynamic paw parameters**

1. **Base of support (fore and hindlimbs)**

| **Hindlimbs** | | | |
| --- | --- | --- | --- |
| **Age** | **Groups** | **Average** | **Standard deviations** |
| **P22** | **C** | 6,18 | 0,89 |
|  | **Pb** | 2,32 | 0,55 |
|  | **Pb+ CurI** | 7,08 | 0,56 |
| **P40** | **C** | 9,07 | 0,78 |
|  | **Pb** | 10,27 | 1,11 |
|  | **Pb+ CurI** | 9,10 | 1,09 |
| **P60** | **C** | 10,23 | 1,09 |
|  | **Pb** | 12,14 | 0,91 |
|  | **Pb+ CurI** | 10,09 | 1,80 |

| **Forelimbs** | | | |
| --- | --- | --- | --- |
| **Age** | **Groups** | **Average** | **Standard deviations** |
| **P22** | **C** | 3,43 | 0,93 |
|  | **Pb** | 1,55 | 0,40 |
|  | **Pb+ CurI** | 3,70 | 0,60 |
| **P40** | **C** | 4,90 | 1,37 |
|  | **Pb** | 6,36 | 1,21 |
|  | **Pb+ CurI** | 5,39 | 1,02 |
| **P60** | **C** | 6,81 | 1,42 |
|  | **Pb** | 9,06 | 1,45 |
|  | **Pb+ CurI** | 7,721 | 1,55 |

(B) **Stride length (fore and hindlimbs)**

| **Hindlimbs** | | | |
| --- | --- | --- | --- |
| **Age** | **Groups** | **Average** | **Standard deviations** |
| **P22** | **C** | 4,20 | 0,02 |
|  | **Pb** | 3,64 | 0,03 |
|  | **Pb+ CurI** | 4,05 | 0,10 |
| **P40** | **C** | 3,95 | 0,08 |
|  | **Pb** | 4,54 | 0,007 |
|  | **Pb+ CurI** | 3,90 | 0,03 |
| **P60** | **C** | 4,61 | 0,13 |
|  | **Pb** | 4,25 | 0,08 |
|  | **Pb+ CurI** | 4,72 | 0,17 |

| **Forelimbs** | | | |
| --- | --- | --- | --- |
| **Age** | **Groups** | **Average** | **Standard deviations** |
| **P22** | **C** | 4,30 | 0,025 |
|  | **Pb** | 3,73 | 0,032 |
|  | **Pb+ CurI** | 4,20 | 0,10 |
| **P40** | **C** | 3,96 | 0,08 |
|  | **Pb** | 4,61 | 0,007 |
|  | **Pb+ CurI** | 3,84 | 0,03 |
| **P60** | **C** | 4,68 | 0,13 |
|  | **Pb** | 4,36 | 0,08 |
|  | **Pb+ CurI** | 4,73 | 0,18 |
